# Digital Twin-Driven Human Robot Collaboration Using a Digital Human

**DOI:** 10.3390/s21248266

**Published:** 2021-12-10

**Authors:** Tsubasa Maruyama, Toshio Ueshiba, Mitsunori Tada, Haruki Toda, Yui Endo, Yukiyasu Domae, Yoshihiro Nakabo, Tatsuro Mori, Kazutsugu Suita

**Affiliations:** 1 National Institute of Advanced Industrial Science and Technology, Koto-ku, Tokyo 135-0064, Japan; t.ueshiba@aist.go.jp (T.U.); m.tada@aist.go.jp (M.T.); haruki-toda@aist.go.jp (H.T.); y.endo@aist.go.jp (Y.E.); domae.yukiyasu@aist.go.jp (Y.D.); nakabo-yoshihiro@aist.go.jp (Y.N.); 2 Toyota Motor Corporation, Toyota 471-8573, Japan; tatsuro_mori@mail.toyota.co.jp (T.M.); kazutsugu_suita@mail.toyota.co.jp (K.S.)

**Keywords:** digital twin, human–robot collaboration, digital human, scheduling, ergonomics

## Abstract

Advances are being made in applying digital twin (DT) and human–robot collaboration (HRC) to industrial fields for safe, effective, and flexible manufacturing. Using a DT for human modeling and simulation enables ergonomic assessment during working. In this study, a DT-driven HRC system was developed that measures the motions of a worker and simulates the working progress and physical load based on digital human (DH) technology. The proposed system contains virtual robot, DH, and production management modules that are integrated seamlessly via wireless communication. The virtual robot module contains the robot operating system and enables real-time control of the robot based on simulations in a virtual environment. The DH module measures and simulates the worker’s motion, behavior, and physical load. The production management module performs dynamic scheduling based on the predicted working progress under ergonomic constraints. The proposed system was applied to a parts-picking scenario, and its effectiveness was evaluated in terms of work monitoring, progress prediction, dynamic scheduling, and ergonomic assessment. This study demonstrates a proof-of-concept for introducing DH technology into DT-driven HRC for human-centered production systems.

## 1. Introduction

The advent of Industry 4.0 [1] has led to the introduction of the digital twin (DT) [2]: A physical product in real space and a corresponding virtual product in virtual space that are connected by data and information. DTs have been utilized in various phases of the production life cycle: design, manufacturing, service, and retirement [2]. A DT corresponds one-to-one with its physical twin, it can simulate the physical twin’s behavior in virtual space exactly, it responds to the physical twin with relatively low latency, and changes to the DT or physical twin affect the other. Thus, a DT offers (1) individualized, (2) high-fidelity, (3) real-time, and (4) controllable performance [3]. It is important that the physical object and the virtual twin are bidirectionally connected, so that not only is the physical object changed based on the virtual twin, but changes in the physical object are also reflected in the virtual twin [4]. DTs are commonly applied in production management [5,6,7]. For example, Oyekan et al. [5] proposed a DT-based fan-blade maintenance system, where the detailed shape of the fan-blade surface is reflected in the DT based on real-time surface measurement by an RGB-depth (RGB-D) camera, and a grinding robot is controlled by the process being simulated with the DT. Human–robot collaboration (HRC) has been introduced to realize safe, rapid, and flexible manufacturing [8,9], and DT-driven HRC has recently emerged as a more reliable and effective approach [10,11]. In a DT-driven HRC system, physical objects such as production facilities and individual robots are reflected in the virtual space as exactly as possible. Real-time analysis and simulation can then be conducted with the DT for safety monitoring and production management. For example, Dröder et al. [12] proposed a DT-driven HRC system for dynamic planning of a safe path for a robot, where the robot and worker are reflected in virtual space by using an RGB-D camera and machine learning. However, previous research has not sufficiently considered human factors for DTs and DT-driven HRC [3]. Considering human movements and ergonomic factors can help the DT monitor the working progress and manage the health and physical loads of workers. Ergonomic indices that are easy to calculate and represent the trunk load are typically used in industrial fields [13], such as the rapid upper limb assessment (RULA) [14] and rapid entire body assessment (REBA) [15]. Such indices can be calculated from the joint angles and positions, body weight, and external forces (e.g., the weight of the held object). However, their applicability decreases as the complexity of the work operation increases. In contrast, the digital human (DH) concept is applicable to detailed analysis of any working posture because the kinematics and dynamics in virtual space can be used to estimate physical loads, such as joint torques [16]. Thus, introducing the DH to DT-driven HRC can potentially realize human-centered production management where an individual worker’s movements, capacity, and physical load are reflected and simulated by the DH in a virtual space. Although this approach has great potential to be used in conjunction with various motion analysis and ergonomic evaluation technologies, to the best of our knowledge, the DH concept and DT-driven HRC including the whole-body motion and worker dynamics have not been integrated to date.

**Figure 1:**
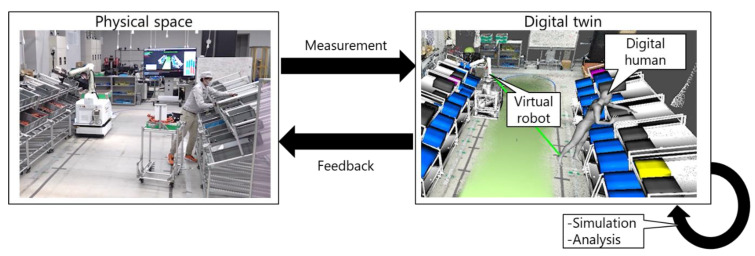
Digital twin in robot, human, and production settings.

In this study, we present a proof-of-concept of the DH-integrated DT-driven HRC system to improve production efficiency and ergonomic evaluation. In our integrated approach, the DH was used to measure worker motions and simulate the working progress and physical load. Further, the proposed system was applied to a parts-picking scenario in a factory environment to demonstrate its applicability to real-world settings. As shown in Figure 1, in the proposed system, the robot and worker movements are reflected by the DT. The proposed system consists of DH, virtual robot, and production management modules. The DH module enables real-time measurement of the full-body motion of the worker, motion analysis, and ergonomic assessment. The virtual robot module contains the robot operating system (ROS), and it controls the robot and acquires its sensor data. Therefore, the DT includes both the worker and robot. The production management module monitors and predicts the working progress for dynamic scheduling under ergonomic constraints and real-time support of the worker. As a demonstration, the proposed system was applied to a parts-picking scenario in a factory environment. The performances of the prediction, dynamic scheduling, and ergonomic constraints were validated with test data imitating various types of workers. This study is a proof-of-concept for introducing a DH into the DT-driven HRC system to enhance the production efficiency and ergonomic evaluation.

The key concepts of the DT include (1) individualized, (2) high-fidelity, (3) real-time, and (4) controllable performance [3]. According to these concepts, the proposed system is organized as follows:

1. The working capability and actual movements in real space are considered by measuring individual robot and worker behaviors.
2. The robot behavior and full-body posture are reflected in the virtual space. The DH module enables further analysis for the worker, such as picking detection and ergonomic evaluation.
3. The virtual and real spaces are connected via real-time measurements, wireless communication, and feedback systems.
4. The changes in the robot and worker in the real space are immediately reflected to the virtual twin. The dynamic scheduling results are immediately presented to the robot and worker to improve the production efficiency and the physical load of the worker.

This study makes the following contributions:

- DH integration: A DH is applied to DT-driven HRC to realize the real-time monitoring, prediction, and ergonomic assessment based on the full-body dynamics of workers.
- Demonstration for a picking scenario: The proposed system was applied to a picking scenario to demonstrate the advantages of DT-driven HRC. The worker and robot picked parts as instructed by the production management module while moving over a wide area.

## 2. Related Works

### 2.1. Human Representation in Research on Human–Robot Collaboration

**Figure 2:**
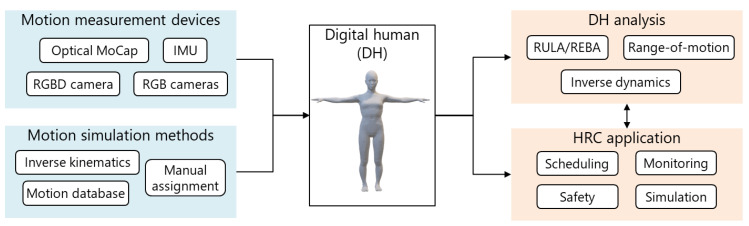
Application of digital humans to human–robot collaboration.

Research on HRC has used various formats to represent workers. The worker representation is especially important for safety monitoring [8]. Michalos et al. [17] proposed an augmented-reality system for displaying the dynamic safe zone to a worker, where the safe zone is changed according to a DT simulation of the robot. However, their DT does not include the worker’s movement, which makes planning safe movements for the robot difficult. Several other studies have focused on capturing worker movements by using an RGB-D camera. Bonci et al. [18] realized collision avoidance by generating an occupancy map acquired using an RGB-D camera mounted on the manufacturing robot. They utilized the acquired point cloud without the semantic labeling of the worker. Nikolakis et al. [19] calculated the nearest-neighbor distance between the simulated robot arm and point cloud of the human user as extracted from an environment-fixed RGB-D camera. Dröder et al. [12] constructed a DT by clustering the point cloud acquired from an RGB-D camera and simulated a collision-free trajectory for the robot. In these studies, the direct use of a point cloud led to safe robot movement even if faulty skeletal recognition occurred. Cheng et al. [20] realized safe motion planning based on using a full-body skeleton to predict the trajectory of workers. They further estimated the current motion class and target object for detailed recognition of worker behavior. Recently, Kanazawa et al. [21] proposed a motion prediction-based algorithm for safe robot motion planning, where the worker’s body center and hand positions are predicted by using a Gaussian mixture model. Finally, Malik et al. [10] utilized the DH model for several ergonomic assessments during working. Thus, workers have been represented as point clouds, joint positions, full-body skeletons acquired by cameras or inertial measurement units (IMUs), the class of the current motion, and DHs. According to Fera et al. [22], because the DH contains both skeleton and volumetric body segments, it is potentially applicable to safety monitoring, motion analysis, and ergonomic assessment. Therefore, the proposed system employs a DH for human representation, and the full-body motion of the worker is assigned to the DH in real time. As shown in Figure 2, using the DH as a common representation of the worker enables the system to connect with various human measurement and analysis modules for a given production scenario.

### 2.2. Improvement in Production Efficiency by Human–Robot Collaboration

HRC and DTs have been applied to improving the production efficiency of flexible manufacturing [10]. Monitoring workers enables dynamic scheduling based on the actual progress [22]. For example, Cheng et al. [20] proposed an HRC system that performs dynamic scheduling by using an RGB-D sensor to recognize the current actions of workers. They confirmed that dynamic scheduling led to improved time efficiency. Lv et al. [23] proposed a comprehensive DT-based HRC assembly system where physical entities such as the robot, facilities, and worker movements are mapped to virtual space. The safety module and dynamic task allocation were performed according to dynamic changes in the DT of the assembly environment. Zhang et al. [24] proposed a dynamic scheduling algorithm based on worker capability, where they employed the hand speed and its variance, assembly accuracy, and variance of the hand trajectory as the human performance index. This enables dynamic scheduling adapted to the performance of an individual worker. Bilberg and Malik [25] used the measured cycle time to evaluate the skill index of a worker, and the assembly tasks were allocated to balance the workloads of the robot and worker. Their consideration of the variability in worker capability effectively improved the progress prediction.

In this study, dynamic scheduling was performed to reduce the delay in progress between the robot and worker while allocating or deallocating tasks with low or high physical loads, respectively. This is realized by estimating the current working progress, future working progress, and physical load during working. The current working progress is recognized by the motion analysis of the worker. The future progress is predicted on the basis of the actual cycle time of the worker. The physical load is estimated by calculating the kinematics and dynamics of the DH.

### 2.3. Ergonomic Assessment in Industry Fields

Ergonomic assessment is an important issue for industrial workers [1,5]. Although both physical and cognitive loads must be assessed [26], this study focused on the physical aspects (i.e., ergonomics). Oyekan et al. [27] revealed human reactions to robot motions by collecting human characteristics in the factory DT integrated with the DH simulation. Most studies on ergonomic assessment in the industry have utilized quick ergonomic indices such as RULA [14], REBA [15], the Ovako working posture analysis system (OWAS) [28], the National Institute for Occupational Safety and Health (NIOSH) lifting equation [29], and the occupational repetitive actions (ORCA) checklist [30]. Maderna et al. [31] proposed a kitting scheduling algorithm to reduce both the cycle time and physical load of the worker. The physical load is scored according to RULA and REBA for the full-body skeleton acquired by the RGB-D camera. The time-integrated value of REBA is employed as the physical load index in the literature [32], where the task planner proposed the task sequence that removes the burdensome posture scored by the REBA index. Greco et al. [33] constructed a DT of the factory layout and worker movement. They applied the OWAS, NIOSH, and ORCA checklists to evaluate the layout in terms of ergonomics. Although limited in their applicability to a given task and posture, these indices allow for quick assessment of the ergonomics. Meanwhile, Baskaran et al. [34] indicated the need for integrating DT with DH technology. Reflecting the worker as the DH enables ergonomic assessment of various physical properties (e.g., body height, weight, and range of motion), motion simulation in the design phase, and motion analysis in the manufacturing phase. For example, Malik et al. [10] utilized a DH simulation for ergonomic assessment of the workspace layout based on visual and grasp analyses. Furthermore, Menychtas et al. [35] demonstrated the effectiveness of joint torque analysis for evaluating the physical load on industrial workers.

In this study, a DH was employed for a consistent and comprehensive representation of the worker in the DT-driven HRC framework. This enabled the ergonomic assessment of not only the REBA and OWAS but also joint torques. Thus, in contrast to previous studies, dynamic scheduling using joint torques was realized in this study, where the joint torque was estimated according to the kinematic and dynamic analyses of the DH.

As introduced so far, individual studies have been performed to improve human representation, production efficiency using DT and HRC, and the accuracy of ergonomic assessment. However, to the best of the authors’ knowledge, no DT-driven HRC system has been reported with an enhanced production efficiency based on a detailed physical load assessment such as joint torque using DH. Such integration research and proof-of-concept with a realistic production scenario are essential for the development of DT research involving humans.

## 3. Method

### 3.1. Overview

**Figure 3:**
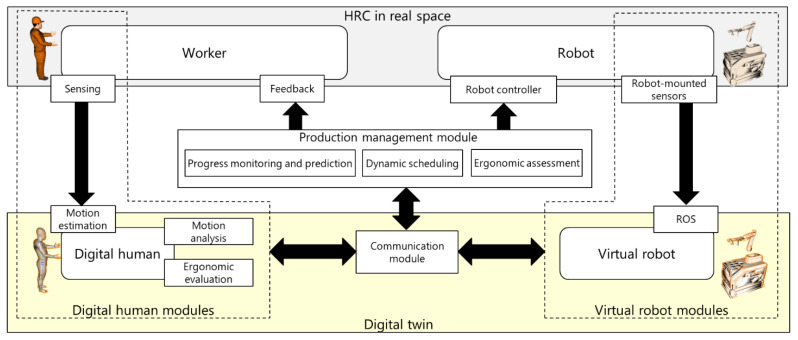
Digital twin-driven human–robot collaboration framework.

Figure 3 shows the proposed DT-based HRC framework. The DT consists of the DH and virtual robots generated using real-time measurements of the worker and robot. The sensor submodule and robot-mounted sensors are the interfaces used to map the physical worker and robot to virtual space. In contrast, the feedback submodule and robot controller are the interfaces used to change the behaviors of the worker and robot based on commands of the production management module. The communication module realizes bidirectional wireless communication among the DH, virtual robot, and production management modules. Finally, the production management module monitors and predicts the progress for dynamic scheduling and ergonomic assessment based on the DT. The production management, DH, and virtual robot modules are independent and communicate via a wireless network because the integrated system adapts to various production scenarios by replacing or customizing each module, where the workplace layout, manufacturing robot and control modules, worker movements, and production management strategy change frequently. The details are described in the following subsections.

### 3.2. Digital Twin of the Worker

As shown in Figure 3, the DH module includes submodules for sensing, motion estimation, motion analysis, and ergonomic assessment. Worker movements are captured via the sensing submodule, and the motion estimation submodule reconstructs the full-body posture by using the DH model. The DH model is further used for motion analysis such as picking detection and for ergonomic assessment such as joint torque analysis.

#### 3.2.1. Digital Human Model

**Figure 4:**
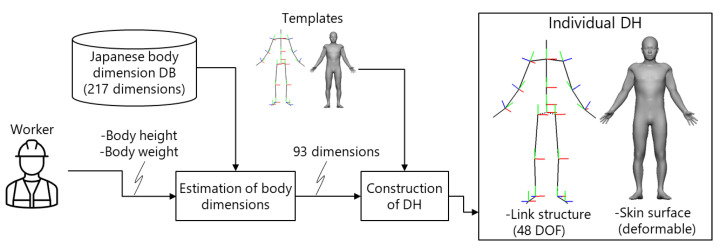
Construction of the digital human model.

As shown in Figure 4, the DH model consists of a link structure with 48 degrees of freedom and a skin surface that deforms with changes in the joint angles [36,37]. The 93 body dimensions and segment mass properties of the DH model are estimated to reflect the body height and weight of the worker based on principal component analysis of information taken from the body dimension database for Japanese people [38]. The details are described in [36,37].

#### 3.2.2. Sensing Submodule

The types and combination of sensing submodules are determined using the production scenario: the target measurement space; environmental conditions such as light, occlusions, and magnetic disturbance; and the acceptability of a wearable sensor. Previous studies utilized an RGB-D camera to acquire the point cloud and body skeletal data. However, this sensor is limited to situations where the worker moves in a limited area, i.e., within the field of view. In this study, marker-based optical motion capture (OptiTrack [39]) was employed as the sensing submodule, where 3D marker positions are captured by multiple cameras arranged in the environment.

#### 3.2.3. Motion Estimation

**Figure 5:**
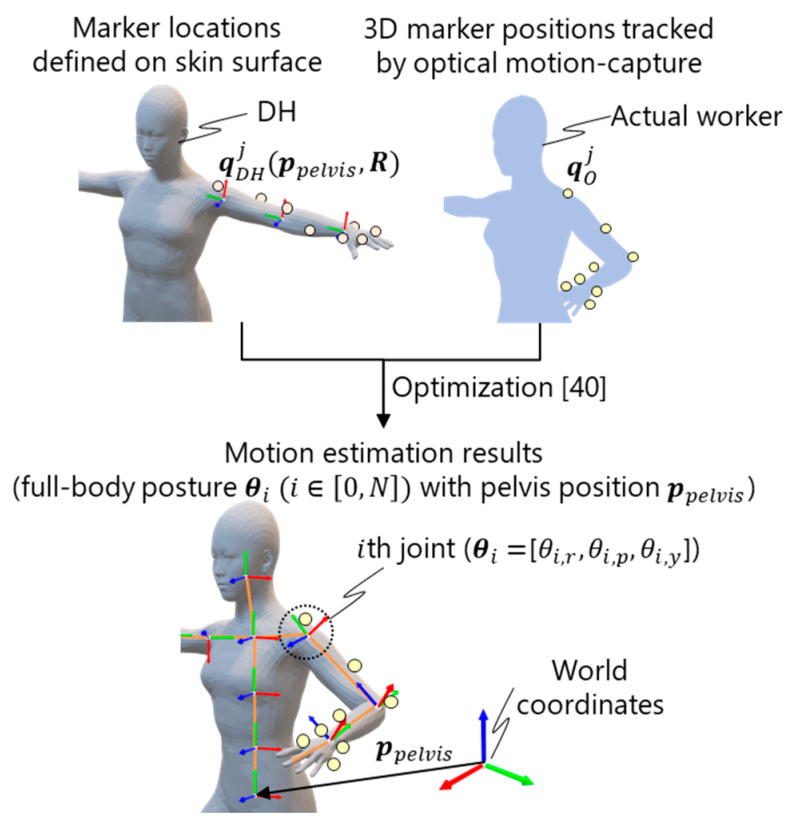
Optimization-based motion estimation for marker-based motion capture [40].

The full-body motion of the worker is estimated on the basis of the sensor data from the sensing submodule. An optimization-based motion estimation algorithm [40] is used, where the posture of the DH is estimated to fit to the sensor data under range-of-motion constraints. As shown in Figure 5, the markers defined on the skin surface are fitted to the 3D marker positions tracked via the optical motion capture by minimizing an objective function:

(1) $$F\left(p_{pelvis},\;R\right)=w_{M}F_{M}\left(p_{pelvis},\;R\right)+w_{R}F_{R}\left(R\right),$$

where $p_{pelvis}$ and $R=\left[\theta_{0},\;\ldots ,\theta_{i},\;\ldots ,\theta_{N}\right]$ are the design variables. $p_{pelvis}$ is the 3D pelvis position, and R includes the roll, pitch, and yaw angles of the ith joint $\theta_{i}=\left[\theta_{i,r},\;\theta_{i,p},\;\theta_{i,y}\right]$. N represents the number of joints. $w_{M}$ and $w_{R}$ are weight parameters. As shown in Figure 5,$\;F_{M}\left(p_{pelvis},\;R\right)$ represents the Euclidean distance between the tracked 3D marker position $q_{O}^{j}$ and predefined points on the skin surface $q_{DH}^{j}\left(p_{pelvis},\;R\right)$, and it is calculated as follows:

(2) $$F_{M}\left(p_{pelvis},\;R\right)=\sum_{j\in \left[0,\;\;M\right]}||q_{O}^{j}-q_{DH}^{j}{(p_{pelvis},\;R)||}^{2},$$

where M represents the number of tracked markers. $q_{DH}^{j}\left(p_{pelvis},\;R\right)$ is obtained by assigning $p_{pelvis}$ and R to the DH, i.e., forward kinematics. $F_{R}\left(R\right)$ is the penalty function to ensure that the joint range of motion is satisfied, and it is calculated as follows:

(3) $$F_{R}\left(R\right)=\sum_{i\in \left[0,N\right]}\sum_{k\in \left\{r,\;p,\;\;y\right\}}\{\begin{array}{cc} \rho \left({\left(\theta_{i,k}-\varphi_{i,k}^{min}\right)}^{2}\right) & \left(\theta_{i,k}<\varphi_{i,k}^{min}\right)\\ \rho \left({\left(\theta_{i,k}-\varphi_{i,k}^{max}\right)}^{2}\right) & \left(\theta_{i,k}>\varphi_{i,k}^{max}\right)\\ 0 & \left(otherwise\right) \end{array},$$

where $\varphi_{i,k}^{min}$ and $\varphi_{i,k}^{max}$ represent the minimum and maximum angles of the ith joint, i.e., range-of-motion, and ρ(x) denotes the Cauchy loss function, ρ(x)=log(1+x).

#### 3.2.4. Motion Analysis

**Figure 6:**
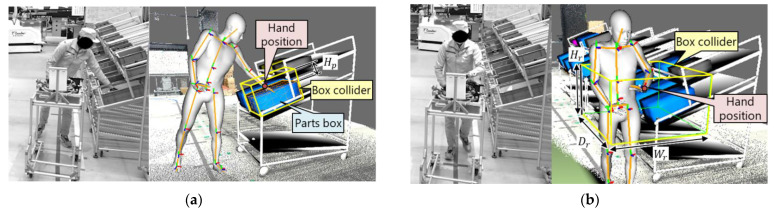
Detection of (**a**) part picking and (**b**) release. Box colliders are created for the parts box and trolley.

The reconstructed full-body motion of the DH can be utilized for several motion analyses. In this study, it was applied to picking detection. As shown in Figure 6, the DT includes details of the picking environment such as the racks and parts box. Thus, picking detection can be realized by detecting the collision between the hand position of the DH and the box space, which is $H_{p}$ [mm] higher than the parts box (Figure 6a). The height increment $H_{p}$ was set to $H_{p}=350$. As shown in Figure 6b, when a worker picks a part, they store the part on a trolley. The part release motion is recognized by detecting the collision between the DH and box space representing the storage, whose height $H_{r}$, width $W_{r}$, and depth $D_{r}$ were set to $H_{r}=300$, $W_{r}=700$, and $D_{r}=600$, respectively. The detection of the part picking and release is used for progress monitoring and prediction.

#### 3.2.5. Ergonomic Assessment

**Figure 7:**
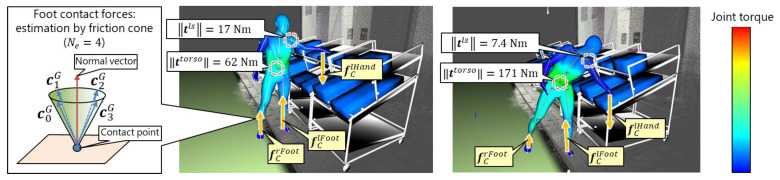
Joint torque estimation for parts picking from upper (**left**) and lower (**right**) shelves. The foot contact forces $f_{C}^{lFoot}$ and $f_{C}^{rFoot}$ are estimated using the friction cone. The joint torque estimation result is visualized using a heat map. The friction cone is approximated by $c_{i}^{G}\;\left(i\in \left[0,3\right]\right)$ when $N_{e}$ is set to $N_{e}=4$.

Once the full-body motion of the worker is reconstructed, several ergonomic assessment methods can be applied. This study focused on joint torque analysis, which quantifies physical load considering full-body posture, body shape and weight, and external forces to a worker. As shown in Figure 7, the joint torque analysis is based on inverse dynamics analysis implemented in the in-house DH software platform DhaibaWorks [41]. The joint torque is obtained using an optimization method [41] under the assumption that the gravity, inertial, and contact forces are in equilibrium:

(4) $$m_{G}g+f_{C}^{G}+f_{K}^{G}+f_{H}^{G}=0.$$

$m_{G}$ and g are the mass of the body segment G and gravity acceleration vector, respectively. $f_{C}^{G}$, $f_{K}^{G}$, and $f_{H}^{G}$ represent the contact forces for segment G, joint reaction forces between segment G and its parent segment, and the joint reaction forces between segment G and its child segment.

In addition, the moments are also assumed to be in equilibrium:

(5) $$r_{cog}^{G}\times m_{G}g+t_{C}^{G}+t_{K}^{G}+t_{H}^{G}=0,$$

where $t_{C}^{G}$, $t_{K}^{G}$, and $t_{H}^{G}$ represent the torques for segment G caused by the forces $f_{C}^{G}$, $f_{K}^{G}$, and $f_{H}^{G}$, respectively. $r_{cog}^{G}$ represents the position vector of the center of gravity of the segment G. The forces $f_{K}^{G}$ and torques $t_{K}^{G}$ can be written as follows:

(6) $$f_{K}^{G}=\sum_{j=0,1,2}x_{K,\;j}^{G}e_{j},$$

(7) $$t_{K}^{G}=\underset{j=0,1,2}{\sum }x_{K,\;j+3}^{G}e_{j},$$

where x∈X represents the optimization variables that describe the forces and torques. $e_{0}$, $e_{1}$, and $e_{2}$ represent the unit vectors $e_{0}=$ [1, 0, 0], $e_{1}=\left[0,\;1,\;0\right]$, and $e_{2}=\left[0,\;0,\;1\right]$, respectively. Finally, all forces and torques are estimated by minimizing the sum of the squares of the contact forces and joint torques:

(8) $$F_{t}\left(X\right)=\sum_{x\in X}{\left(\omega_{x}\;x\right)}^{2},$$

where $\omega_{x}$ is the weight coefficient for x and has a specified constant value ($\omega_{x}=1$).

Figure 7 shows the external forces for the joint torque estimation. In this study, the foot reaction forces $f_{C}^{rFoot}$ and $f_{C}^{lFoot}$ and the hand force $f_{C}^{lHand}=w\left(p_{k}\right)g$ were applied for the right foot (G=rFoot), left foot (G=lFoot), and left hand segments (G=lHand), where $w\left(p_{k}\right)$ represents the weight of part $p_{k}$. As shown in Figure 7, because $f_{C}^{G}$ for the body segments G∈[rFoot, lFoot] is difficult to measure in a factory setting, these forces are defined according to the friction cone:

(9) $$f_{C}^{G}=\sum_{i=\left[0,\;N_{e}\right]}x_{C,\;i}^{G}\;c_{i}^{G},$$

(10) $$t_{C}^{G}=\underset{j=0,1,2}{\sum }x_{C,N_{e}+j}^{G}\;e_{j},$$

where $c_{i}^{G}$ is the unit vector dividing the conical side of the friction cone into $N_{e}$ surfaces.

The friction cone is created so that its top vertex and central axis correspond to the contact position and normal vector, respectively. Finally, all joint torques, joint reaction forces, and contact forces are estimated by solving Equation (8) under the constraints given by Equations (4), (5), (9) and (10). The joint torques are calculated for when the worker picks a part with the hand force $f_{C}^{lHand}$. In this study, the joint torques of the torso $t_{}^{torso}\left(p_{k}\right)$ and left shoulder $t_{}^{ls}\left(p_{k}\right)$ when picking the part $p_{k}$ were calculated and used as the ergonomic constraint for the dynamic scheduling.

### 3.3. Digital Twin of the Robot

**Figure 8:**
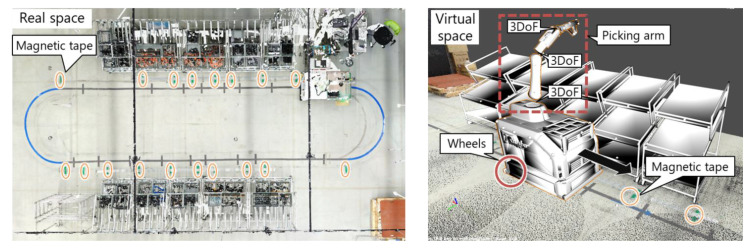
Digital twin of the robot. Magnetic tape in real space is represented in the virtual space.

The picking by the robot is controlled by the ROS. As shown in Figure 8, the current position and orientation of the robot are estimated according to the integration of the rotation velocities of the wheels (i.e., odometry). Magnetic tape is arranged on the floor to reflect positions in the virtual environment of the ROS. The accumulated localization error is corrected by relocating the virtual robot at the magnetic tape in the virtual space when the actual magnetic tape is detected. The posture of the robot arm is reconstructed by solving the forward kinematics from the joint angles of the robot arm. Given the ID of a target part, the picking robot moves and stops in front of the parts box and tries to pick the corresponding part from the box. The result of the part picking (i.e., success or failure) is then recognized. If the magnetic sensor detects a magnetic disturbance after a part is picked, the picking process is considered successful. Otherwise, the picking process has failed, so the robot tries to pick the part again until successful.

### 3.4. Communication Module

The DTs of the worker and robot are integrated by the bidirectional wireless communication module, which enables the development of the virtual robot and DH modules on different platforms. The real-time publish subscribe [42] is used as the communication protocol, and the data distribution service (DDS) [43] is employed as middleware in eProsima FirstDDS [44]. The virtual robot module initially publishes the mesh data of the robot and picking environment. It then publishes the current position and orientation of the robot, the target parts, and the picking state (before picking, success, or failure). The production management module determines the target parts for the worker and robot to pick. Then, it publishes the IDs of the target parts to the virtual robot module when the picking is successful. Thus, the proposed system integrates the DTs of the worker (i.e., DH module) and robot (i.e., virtual robot module). This enables the production management module to update the robot behavior based on the dynamic scheduling results.

### 3.5. Production Management Module

#### 3.5.1. Cycle and Parts Definition

The part definition (part ID $p_{k}$, weight $w\left(p_{k}\right)$, picking time $u\left(p_{k}\right)$, and picking attribute $r\left(p_{k}\right)$), part layout (position $L\left(p_{k}\right)$ and amount $A\left(p_{k}\right)$), cycle pattern $C_{i}=\left\{p_{j}\right\}$, and corresponding picking allocation $R_{i}=\left\{r_{j}\right\}$ are imported into the production management module. The picking attribute $r\left(p_{k}\right)\in \left\{worker,\;robot,\;any\right\}$ indicates whether the part $p_{k}$ can be picked only by the robot ($r\left(p_{k}\right)=robot$), only by the worker ($r\left(p_{k}\right)=worker$), or by both ($r\left(p_{k}\right)=any$). $C_{i}$ indicates the picking sequence of parts for the ith cycle. $r_{j}\in \left\{worker,\;robot\right\}$ indicates whether the robot ($r_{j}=worker)$ or worker ($r_{j}=robot$) takes the part $p_{j}$. Dynamic scheduling is performed when a delay is detected, and the production management module changes the picking allocation $r_{j}$ to recover the schedule.

#### 3.5.2. Progress Monitoring and Prediction

**Figure 9:**
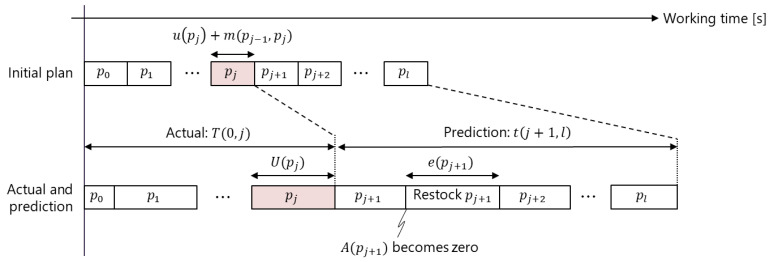
Working time prediction. $p_{j}$ is the part currently being picked. The upper row represents the working time calculation for the initial plan. The lower row represents the calculation of the actual working time T(0, j) and prediction of the future working time t(j+1, l).

As shown in Figure 9, the DT is used to estimate the current progress and predict future progress. The same method is applied for both the robot and worker. The current progress of the worker picking the jth part is calculated as follows:

(11) $$\begin{array}{c} T\left(i,j\right)=\overset{}{\underset{p_{k}\in P\left(i,j\right)}{\sum }}U\left(p_{k}\right)\\ P\left(i,j\right)=\{p_{k}\;|p_{k}\in C_{c},k\in \left[i,j\right],\;r_{k}=r\} \end{array}$$

where T(i,j) is the working time from picking the ith to jth parts in the current cycle $C_{c}$. U(k) is the actual time required to pick the jth part. r∈{worker, robot} is set to r=worker or r=robot when calculating the progress of the worker or robot, respectively. The working speed representing the actual working time relative to the initial plan is calculated as follows:

(12) $$v\left(i,j\right)=T\left(i,j\right)/\left\{\overset{}{\underset{p_{k}\in F\left(i,j\right)}{\sum }}u\left(p_{k}\right)+\overset{}{\underset{p_{k}\in F\left(i,j\right)}{\sum }}m\left(p_{k-1},\;p_{k}\right)\right\}\;F\left(i,j\right)=\{p_{k}\;|p_{k}\in C_{t},k\in \left[i,j\right],\;r_{k}=r\}$$

where v(i,j) is the ratio of the working time to the initial plan from the ith to jth parts in the prediction target cycle $C_{t}$. $m\left(p_{k-1},\;p_{k}\right)$ is the time for moving from $p_{k-1}$ to $p_{k}$ and is calculated as $m\left(p_{k-1},\;p_{k}\right)=d\left(p_{k-1},\;p_{k}\right)/v_{0}$, where $d\left(p_{k-1},\;p_{k}\right)$ and $v_{0}$ represent the distance between the fronts of the k−1th to kth parts and the predefined walking speed, respectively. Finally, the future progress is predicted as follows:

(13) $$t\left(j+1,\;l\right)=v\left(0,j\right)\left\{\overset{l}{\underset{p_{k}\in F\left(j+1,l\right)}{\sum }}u\left(p_{k}\right)+\overset{}{\underset{p_{k}\in F\left(j+1,l\right)}{\sum }}m\left(p_{k-1},\;p_{k}\right)\right\}+\overset{}{\underset{p_{k}\in F\left(j+1,l\right)}{\sum }}e\left(p_{k}\right)\;e\left(k\right)=\{\begin{array}{cc} E & A\left(p_{k}\right)=0\\ 0 & otherwise \end{array}$$

where E represents the time to restock a parts box. Because the proposed system can recognize the picking of $p_{k}$, changes to the amount of parts $A\left(p_{k}\right)\;$can be tracked and predicted when part $p_{k}$ is picked. In this study, the prediction target cycle $C_{t}$ (i.e., the range of prediction) was set to $C_{t}=C_{O}\cup C_{O+1}$ for predicting the current cycle $C_{O}$ and next cycle $C_{O+1}$.

#### 3.5.3. Dynamic Scheduling

To ensure safe and efficient production, dynamic scheduling is performed at picking of jth part when the following equation is satisfied:

(14) $$T_{robot}\left(0,j\right)+t_{robot}\left(j+1,l\right)-\left\{T_{worker}\left(0,j\right)+t_{worker}\left(j+1,l\right)\right\}\;\geq D_{th}$$

where $T_{robot}\left(0,j\right)+t_{robot}\left(j+1,l\right)$ and $T_{worker}\left(0,j\right)+t_{worker}\left(j+1,l\right)$ represent the working times from picking the first to lth parts picking of the robot (r=Robot) and worker (R=Worker), respectively.

The working times comprise the actual working time until the jth part is picked $T_{*}\left(0,j\right)$ and the predicted working time until the lth part is picked $t_{*}\left(j+1,l\right)$. $D_{th}$ is the threshold value representing the acceptable delay time. If Equation (14) is satisfied, this indicates that the worker’s task will be delayed by more than $D_{th}$ (s) compared with that of the robot. Then, the picking allocation $r_{i}$ assigned to the worker (i.e., $r_{i}=worker$) should be changed to $r_{i}=robot$ until Equation (14) is no longer satisfied. First, the set of exchangeable parts $P_{r}$ that is assigned to the worker but can be assigned to the robot is extracted as follows:

(15) $$P_{R}=\{p_{i}\;|\;i\in \left[j+1,\;l\right],\;r_{i}=worker,\;r\left(p_{i}\right)=any\}$$

Then, the parts $p_{e}$ for which $r_{e}$ is changed to $r_{e}=robot$ is selected to minimize the maximum physical load for picking $p_{i}\in P_{r}$:

(16) $$p_{e}=\underset{p_{i}\in P_{r}}{\max}\left\{\underset{}{\max}\left\{\hat{t^{torso}}\left(p_{i}\right),\;\hat{t^{ls}}\left(p_{i}\right)\right\}\right\},$$

where $\hat{t^{torso}}\left(p_{i}\right)\in \left[0,1\right]$ and $\hat{t^{ls}}\left(p_{i}\right)\in \left[0,1\right]$ are the normalized values of the torso and shoulder joint torques $t^{torso}\left(p_{i}\right)$ and $t^{ls}\left(p_{i}\right)$, respectively, when the part $p_{i}$ is picked. The joint torque $t\left(p_{i}\right)$ is normalized as follows:

(17) $$\hat{t}\left(p_{i}\right)=||t\left(p_{i}\right)||/\underset{p_{k}\in C_{c}}{\max}\left\{||t\left(p_{k}\right)||\right\}.$$

Equations (15)–(17) are used to assign the parts $p_{e}\;$with the highest physical load to the robot sequentially until Equation (14) is no longer satisfied.

#### 3.5.4. Feedback Submodule

**Figure 10:**
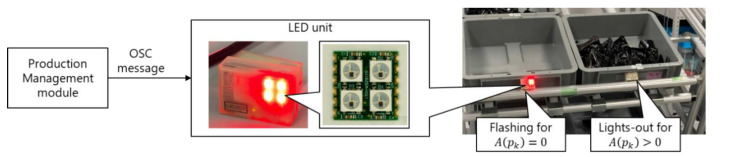
Feedback to the worker.

As shown in Figure 10, the instructions are sent from the production management module to the worker via earphones and the wireless light-emitting diode (LED) submodule. The ID of the next part picked by the worker $p_{n}$ is announced via earphones, where $p_{n}$ is determined by the progress monitoring and dynamic scheduling. In addition, the request to restock the parts box is displayed by the LED submodule. When $A\left(p_{k}\right)$ becomes zero, the LED submodule attached to the box of parts $p_{k}$ flashes. When the parts box is restocked by the worker, then the LED submodule stops flashing. Note that the worker needs to restock the box of parts $p_{k}$ even if it is assigned to the robot (i.e., $r_{j}=robot$).

## 4. Results

### 4.1. Experimental Settings

**Figure 11:**
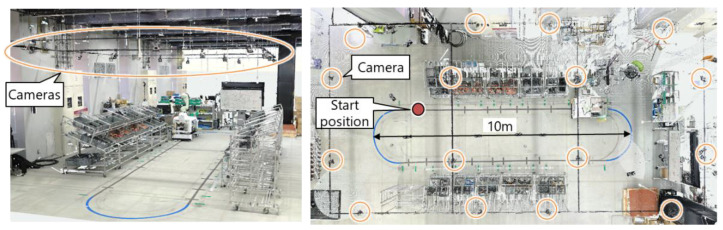
3D laser-scanned point clouds of the picking environment.

Figure 11 shows the picking environment of the demonstration experiment. Sixteen cameras were arranged under the ceiling for optical motion capture. Four parts boxes were located on the rack, and there were 40 parts boxes in total. We tested the proposed system through an online demonstration with an operator and an offline validation with test data.

**Figure 12:**
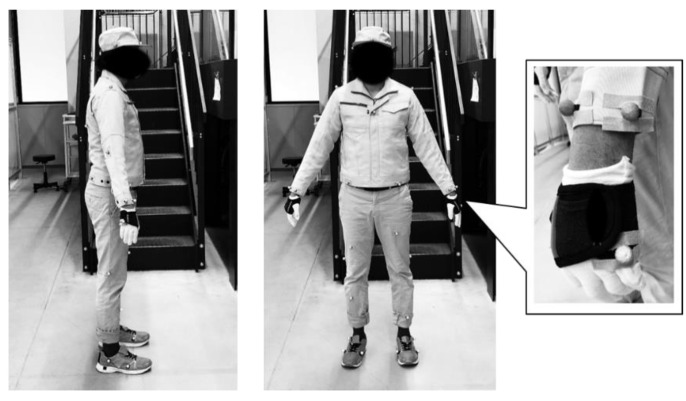
Work cloth with 37 reflective markers.

In the online demonstration, as shown in Figure 12, 37 reflective markers were attached to the clothes, gloves, and shoes of the worker for motion capture. The production management module instructed the robot to start picking half a cycle after the worker started picking. The performance of the proposed system was then evaluated in terms of DT construction and dynamic scheduling. Videos are available as Supplementary Material (Video S1).

In the offline validation, the test data shown in Table 1 was used, where various delay factors were imitated. Condition 1 imitates the worker who delays 1.0 s for each part consistently. Condition 2 imitates the worker who slows down as the cycle progresses. Condition 3 imitates the worker whose delay follows the Gaussian distribution.

### 4.2. Construction of the Digital Twin

**Figure 13:**
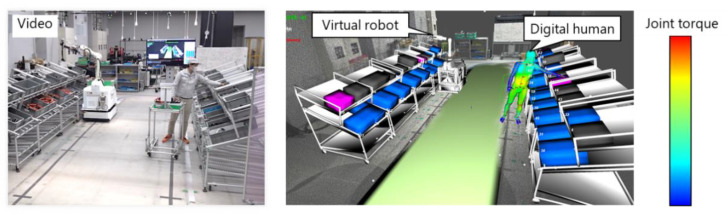
Results for the digital twin construction.

Figure 13 shows the results of the DT construction. The virtual robot and DH accurately reflected the actual postures of the robot and worker, respectively. The color of the DH represents the joint torque estimation results. Table 2 shows the elapsed time of each process. The most time-consuming process is the ergonomic evaluation, which is performed only when the worker picks the target part. Thus, the proposed system ranged from approximately 6 (when picking parts) to 50 fps.

**Figure 14:**
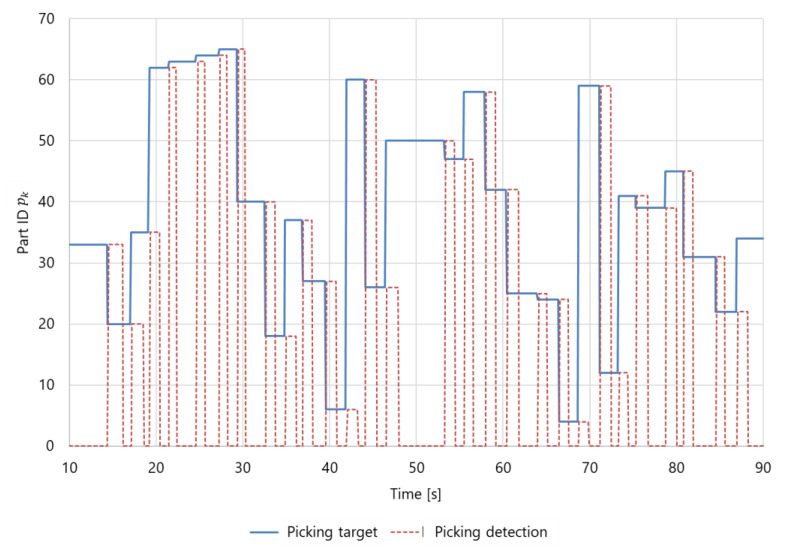
Example detection of a worker picking a part.

Figure 14 shows an example of the picking detection for the worker. The rise of the red dotted line indicates that picking was detected, and the fall of the red dotted line indicates that the held part was released. As shown in the figure, it was confirmed that the picking target part $p_{k}$ of the worker is successfully updated when its picking is detected. At this time, the next part $p_{k}$ is announced to the worker via earphones as described in Section 3.5.4. In the case of the robot, $p_{k}$ is operated via a wireless communication module, as described in Section 3.4. Thus, the picking detection results confirmed that the proposed system could control the part picking.

### 4.3. Online Demonstration with an Operator

#### 4.3.1. Results of Progress Prediction

**Figure 15:**
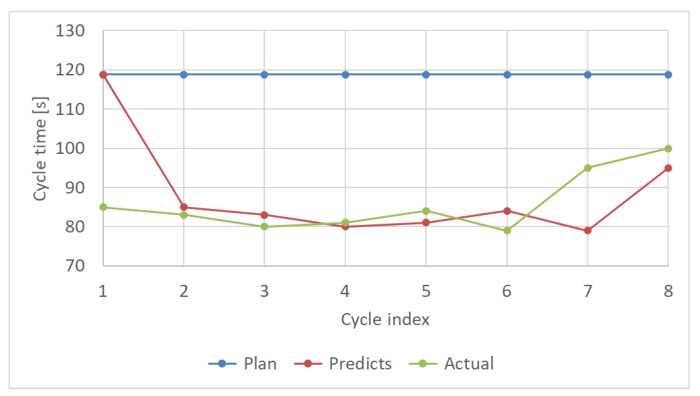
Predicted cycle time of the worker. The prediction is performed at the beginning of each cycle.

Figure 15 shows the results for the progress prediction. The worker performed eight cycles and picked 40 parts per cycle. There was a 50 s difference between the actual cycle time and initial plan before the first cycle. This is because the worker’s performance was not measured by the system. As described in Section 3.5.2, the next cycle time was predicted according to the previous cycle. Therefore, changes in the actual cycle time, such as the difference between the initial plan and the actual cycle time at the 1st cycle, the drops of the actual cycle time at the 6th cycle, and the increases of the actual cycle time at the 7th cycle, was reflected in the predictions at the 2nd, 7th, and 8th cycles, respectively. Table 3 shows the comparison of the difference from the actual cycle time between the initial plan and prediction. The average and standard deviation (μ±σ) of the difference between the initial plan and actual times for eight cycles were 33 ± 7 s, and those of the difference between the prediction and actual time were approximately 9 ± 11 s. The standard deviation of the prediction difference was larger than that of the initial plan because the prediction was performed according to the actual time of the previous cycle. Thus, the standard deviation of the prediction error might be larger as the variability of the actual cycle time increases.

#### 4.3.2. Results for the Dynamic Scheduling

**Figure 16:**
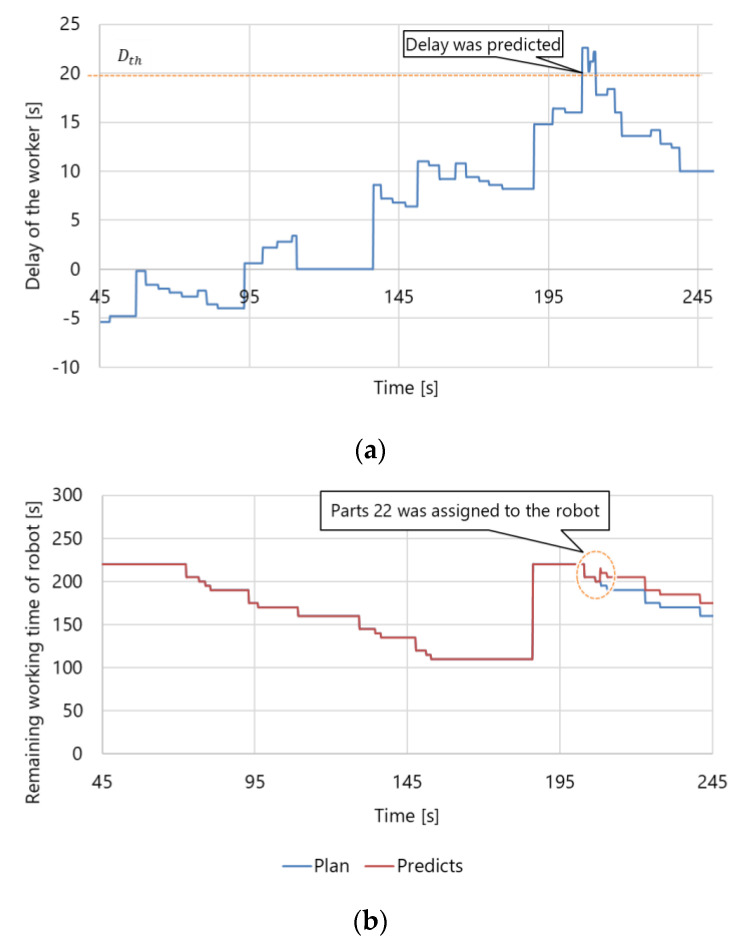
Results for the dynamic scheduling: (**a**) worker delays and (**b**) changes in the predicted working time of the robot. When the delay was detected, the dynamic scheduling was performed so that the delay becomes smaller than the threshold $D_{th}$. Consequently, the remaining working time of the robot was increased from the initial plan.

**Figure 17:**
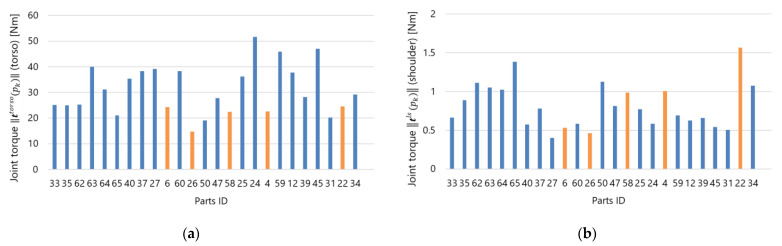

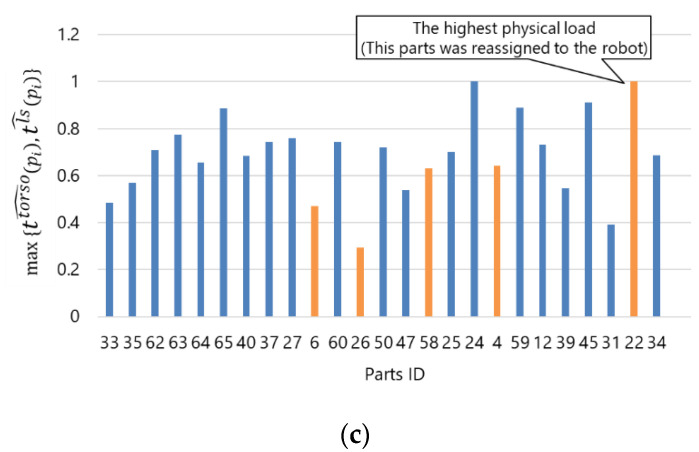
Ergonomic assessment for dynamic scheduling: (**a**) joint torque of the torso, (**b**) joint torque of the left shoulder, and (**c**) estimation of parts with the highest physical loads. Note: Orange: parts that can be assigned to the robot ($r_{k}=worker$, $r\left(p_{k}\right)=any$), Blue: parts that must be picked by the worker ($r_{k}=worker$, $r\left(p_{k}\right)=worker$).

Figure 16 shows the dynamic scheduling results. Figure 16a shows the delay calculated using Equation (13), where the threshold value $D_{th}$ was set to $D_{th}=20$ s. The delay became greater than $D_{th}$ at approximately 200 s. Then, the part $p_{e}$ originally assigned to the worker was assigned to the robot, so the remaining working time of the robot increased as shown in Figure 16b. In this experiment, part 22 (i.e., $p_{e}=22$) was changed from the worker to the robot. This is because part 22 had a greater physical load than the other parts. Figure 17 shows the results for the normalized joint torque estimation. The parts set $P_{r}=\left\{6,\;26,\;58,\;4,\;22\right\}$ could be switched from the worker to the robot. As shown in Figure 17b, part 22 had the highest physical load within $P_{r}$. Therefore, the results confirmed that the proposed system could reduce the delay according to the predicted progress while assigning parts with the highest physical load from the worker to the robot.

### 4.4. Offline Validation with Test Data

**Figure 18:**
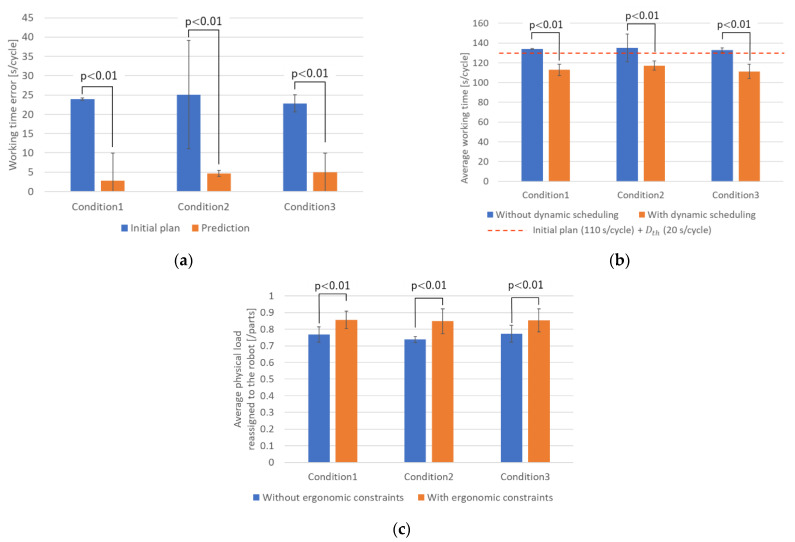
Offline validation with test data: (**a**) the performance of the working time prediction, (**b**) performance of the dynamic scheduling, and (**c**) performance of the ergonomic constraints. The error bars represent the standard deviation.

The proposed system was further validated with the test data shown in Table 1. Figure 18 shows the validation results. We conducted a *t*-test and found a significant difference for all conditions (p<0.01). Figure 18a compares the difference between the actual working time and initial plan, i.e., the worker’s delay, with the difference between the actual working time and prediction using the proposed system. The proposed system could predict the working time more accurately than the initial plan for all the conditions. Moreover, Figure 18b shows the average working time of each cycle. The dynamic scheduling enabled to decrease the worker’s delay significantly, and all delays were kept below the sum of the initial plan (110 s/cycle) and user-specified threshold $D_{th}$ ($D_{th}=20\;s/\mathrm{cycle})$.

Finally, Figure 18c shows the performance of the ergonomic constraints. The average physical loads reassigned to the robot through the dynamic scheduling with ergonomic constraints were significantly larger than those through the dynamic scheduling without ergonomic constraints. From these results, it was confirmed that the proposed system decreases the worker’s delay through the dynamic scheduling while changing the assignment of the parts with high physical load to the robot from the worker.

## 5. Discussion

The proposed system was tested through the online demonstration and offline validation with the test data. In contrast to previous DT-driven HRC studies [3,8,9], the proposed system realized the DT including the robot and worker who moves over a wide area. Through the online demonstration, we confirmed that the proposed system could perform the dynamic scheduling based on picking detection and the ergonomic evaluation of the worker. The average and standard deviation of the prediction error were approximately 9 ± 11 s. Because the prediction is performed according to the actual time of the previous cycle, the standard deviation of the prediction error might be larger as the variability of the actual cycle time increases. Therefore, the prediction accuracy may decrease in cases where the work speed increases or decreases drastically every cycle, although this may not happen with actual workers. The processing speed of the proposed system ranged from approximately 6 (when picking parts) to 50 fps. Considering that the parts-picking work requires a few seconds, this speed is sufficient to manage dynamic scheduling. However, further verification of the processing speed of the system, moving speed of the robot, and data communication speed is necessary for extending the application of the proposed system to a safety monitoring, where the real-time responsibility must be ensured.

The dynamic scheduling performance was tested, and the proposed system was confirmed to decrease the delay based on the predicted progress by assigning parts with the highest physical load from the worker to the robot. As shown in Figure 17, in contrast to discrete evaluations such as REBA [13], the proposed system can quantify the physical load for each individual movement. It leads the priorities for task allocation to reduce the physical load during dynamic scheduling. The proposed system was validated with test data imitating various types of workers. The dynamic scheduling under the ergonomic constraints kept the worker’s delay below the user-specified threshold while decreasing the physical load of the worker. Therefore, this paper presents a proof-of-concept for introducing a DH into a DT-driven HRC system with regard to production efficiency and ergonomic assessment.

To introduce the proposed concept into actual factories, further research on the human measurement system is necessary. An optical motion capture system [39] was employed as the sensing submodule, and all cameras were arranged under the ceiling to avoid occlusions. This system is highly accurate and widely available, but it is costly compared with other motion capture devices such as IMUs [45] and RGB-D cameras [46]. Moreover, attaching 37 markers to the worker and arranging cameras to avoid occlusion are cumbersome; thus, the environment in which it can be implemented is limited. A multimodal or dual measurement system might be desired in actual factories considering the features of each measurement device, e.g., IMU has drift errors, and the RGB-D is affected by occlusions. Therefore, future work will involve validating the motion estimation, picking detection, and ergonomic assessment performance with different and multiple sensing submodules such as IMU and RGB-D cameras. The proposed system was tested with subjects who do not work in an actual factory. This is sufficient for a proof-of-concept study, but the proposed system must be validated with factory workers working for realistic amounts of time for applications to real factory settings. In the case of an actual factory, human errors such as dropping parts or getting duplicate parts may occur. In addition, the workers do not always work in the correct matter, such as storing multiple parts at once. Dealing with such unintended behavior of the worker is a future issue. To understand and handle the various behaviors of actual workers, the proposed system must be tested with actual workers. In addition, only healthy workers were expected in this study, but actual workers may have diseases such as back pain. For the ergonomic evaluation considering such situations, it is necessary to improve the Equations (16) and (17) by introducing weight parameters reflecting the individual physical and kinematic characteristics. This will also be addressed in future work.

## 6. Conclusions

In this study, a proof-of-concept for introducing a DH into a DT-driven HRC system was presented to enhance production efficiency and ergonomic evaluation. The DT-driven HRC system incorporated the DH to reconstruct the full-body posture for motion analysis and ergonomic assessment. The integration with the DH enabled the detailed ergonomic evaluation such as joint torque analysis. Thus, the dynamic scheduling method based on the worker’s joint torque was proposed. The proposed system was applied to a part-picking scenario, and the contribution of the DH integration was confirmed. In particular, the proposed system can predict the working progress by considering the actual performance of the worker. Further, the dynamic scheduling with joint torque could improve the progress of the worker while decreasing the physical load of the worker. The results confirm that the introduction of the DH into the DT-driven HRC can improve productivity and reduce physical load based on the individual capability of the worker.

This study provided a proof of concept for DT-driven HRC and confirmed its effectiveness. However, there are still some issues that need to be addressed before it can be implemented in actual factories. The optical motion capture system in this study is highly accurate, there are issues in terms of price and installation to use such a system in an actual factory. Thus, the selection of an appropriate measurement system remains an issue. Even when the measurement system is changed according to the production scenario, the proposed work prediction and dynamic scheduling can be applied by reflecting the workers as digital human in the virtual space.

Moreover, further verification of the processing speed is necessary for its use in safety monitoring practices. In this paper, the feedback from the system to the worker included the scheduling and next picking parts. Because the proposed DT-driven HRC framework can recognize the full-body posture of the worker, picking parts, and robot behavior, it will be extended to various applications, such as fall detection, abnormal work detection, and safety monitoring. Such extension will be addressed in our future study.

## Figures and Tables

**Table 1:** Test data for validation. c represents the current cycle index. $N\left(\mu ,\;\sigma^{2}\right)$ represents the Gaussian distribution.

Condition	Picking Speed [s/Parts]	Cycles
Initial plan ^1^	4.4	10
Condition 1	4.4 + 1.0	10
Condition 2	4.4 + 0.2 c	10
Condition 3	4.4 + $d\;\left(d\sim N\left(1.0,\;0.5^{2}\right)\right)$	10

^1^ If the worker follows the initial plan, no delay occurs.

**Table 2:** Elapsed time of the proposed system performed on a laptop computer (CPU: Intel(R) Core(TM) i7-1065G7, RAM: 32GB, GeForce GTX 1650). The background model (3D point clouds in Figure 13) was not rendered.

Process	Elapsed Time [ms] ^1^
Motion estimation	15
Visualizing virtual robot	1
Motion analysis	3
Ergonomic evaluation ^2^	138
Progress monitoring and prediction	1
Dynamic scheduling ^2^	1

^1^ Average value in a cycle including dynamic scheduling. ^2^ Performed only when the worker picks parts.

**Table 3:** Comparison of the initial plan and the prediction.

	Actual Cycle Time [s]	Difference between Actual and the Initial Plan [s]	Difference between Actual and the Prediction [s]
Cycle 1	85	34	34
Cycle 2	83	36	2
Cycle 3	80	39	3
Cycle 4	81	38	1
Cycle 5	84	35	3
Cycle 6	79	40	5
Cycle 7	95	24	16
Cycle 8	100	19	5
μ±σ		33 ± 7	9 ± 11

## Data Availability

Data sharing is not applicable.

## Supplementary Materials

The following are available online at https://www.mdpi.com/article/10.3390/s21248266/s1, Video S1: Digital Twin-Driven Human Robot Collaboration Using a Digital Human.mp4.
